# Comparison of capilary glycemic responses after moderated continuous racing and high-intensity interval training in diabetes type 1 patients

**DOI:** 10.1186/1758-5996-7-S1-A228

**Published:** 2015-11-11

**Authors:** Brenda Bauth Barros Oliveira, Nadjara Silva Viana, William Valadares Campos Pereira, Albená Nunes da Silva

**Affiliations:** 1 Universidade Federal de Minas Gerais/ICB, Brazil

## Background

Variations of glucose levels in patients with diabetes type 1 (DM1) in response to different types of exercise protocols are still unclear. This knowledge would permit a more accurate exercise prescription to these specific subjects and augment the security of its performing.

## Objective

The aim of this study was to compare the glycemic response of DM1 subjects after moderate-intensity continuous running protocol (MI) and high-intensity Interval training (HIIT).

## Materials and methods

Seven men with DM1, aged 26±6.63 yrs., BMI 24±1.99 kg/m2, with DM1 duration of 15±9 yrs., HbA1c 7.76±0.4%, and physically active without historical chronic complications volunteered to this study. In the MI protocol, subjects remained at a constant rate for 30 min to maximum 60% of the estimated heart rate. In the HIIT protocol, individuals ran for 1 min at high intensity (higher than 90% HR) and walked 1 min 5 km/h for 20 min. Samples were collected in two different days, with an interval of 48 h between them. The first day was composed of physical assessment, dietary investigation of the last 24 h and realization of the first protocol exercise. The second day also consisted of food recall for the last 24 h and realization of the second protocol exercise. The protocols were assigned randomly. During exercise protocols, perceived exertion (PSE), heart rate (HR), blood pressure (BP) and capillary glucose levels were collected immediately before and after and 30 min after the test.

## Results

**Figure 1:**
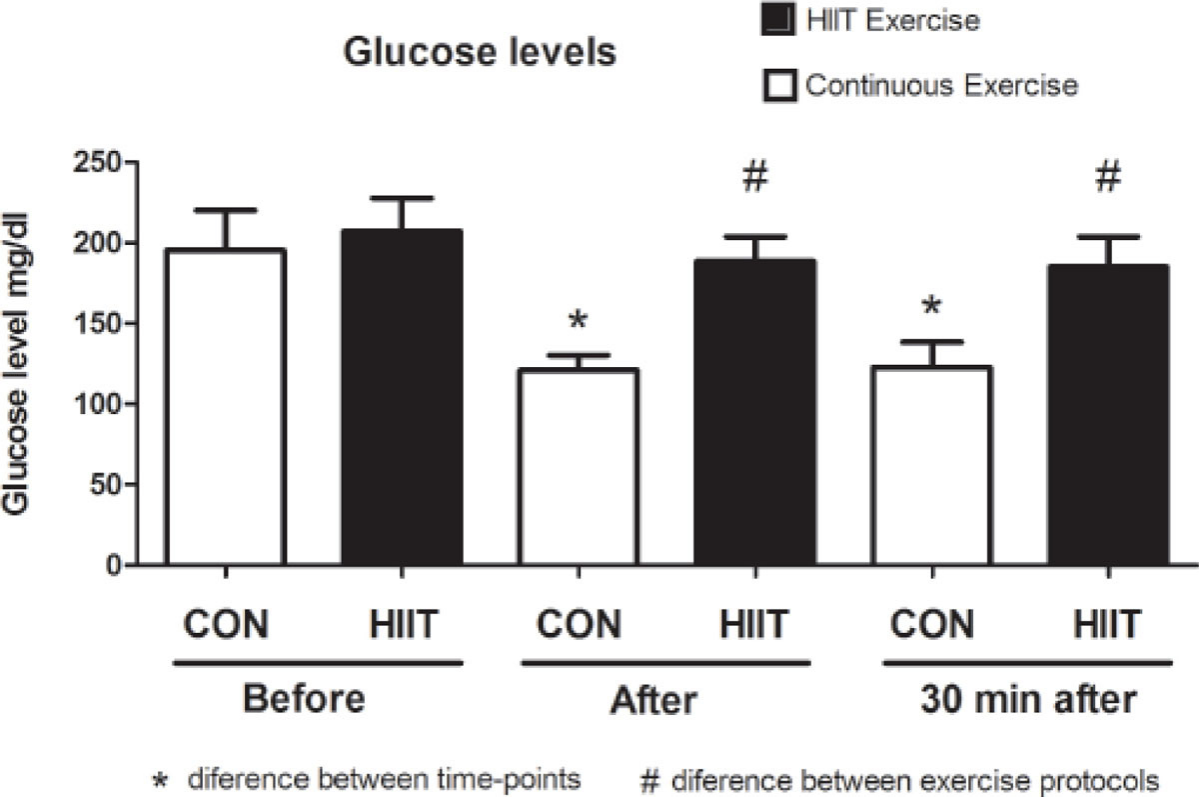
Blood glucose levels in volunteers before, after and 30 minutes after continuous exercise or HIIT exercise.

Both performed protocols resulted in blood glucose level decrease in volunteers. However, HIIT promoted a significantly smaller decrease compared to MI (Figure 1) immediately (p=0.01) and 30 min (p=0.02) after exercise.

## Conclusion

Both exercise protocols induced glucose reduction in peripheral blood. However, the decline in blood glucose was significantly lower in the HIIT protocol when compared to the MI in DM1.

